# Nurses’ and Nursing Assistants’ Experiences With Teleconsultation in Small Rural Long-Term Care Facilities: Semistructured Interview Pilot Study

**DOI:** 10.2196/65111

**Published:** 2024-11-27

**Authors:** Veronique Nabelsi, Marie Chantal Leclerc, Véronique Plouffe

**Affiliations:** 1 Département des sciences administratives Université du Québec en Outaouais Gatineau, QC Canada; 2 Département des sciences infirmières Université du Québec en Outaouais Gatineau, QC Canada; 3 Département des sciences comptables Université du Québec en Outaouais Gatineau, QC Canada

**Keywords:** teleconsultation, long-term care facilities, nursing, nursing practices, workflow optimization, residents, rural, telehealth, Quebec

## Abstract

**Background:**

In Quebec, the shortage of nurses during night shifts compromises the safety and quality of resident care, particularly in small residential and long-term care centers (“Centres d’hébergement et de soins de longue durée”; CHSLDs) located in rural areas. The need to ensure the continuous presence of nurses 24 hours a day in CHSLDs has become more pressing, forcing some facilities to implement exceptional measures such as on-call telephone services to ensure access to a nurse. In light of these challenging circumstances, the Direction nationale des soins et des services infirmiers of Quebec’s Ministère de la Santé et des Services sociaux has rolled out a teleconsultation pilot project.

**Objective:**

This study aims to explore nurses’ and nursing assistants’ experience of integrating teleconsultation during night shifts in rural CHSLDs with ≤50 residents.

**Methods:**

The 6-month pilot project was rolled out sequentially in 3 rural CHSLDs located in 2 administrative regions of Quebec between July 2022 and March 2023. A total of 18 semistructured interviews were conducted with 9 nurses and nursing assistants between February and July 2023.

**Results:**

Participants’ experiences revealed that teleconsultation provided significant added value by improving clinical, administrative, and organizational practices. Some practices remained unchanged, indicating stable workflows. Workflow optimization through an expanded scope of practice ensured efficient and safe continuity of care. Enhanced collaboration between nurses and nursing assistants led to improved care coordination and communication. The leadership played a significant role in clarifying professionals’ roles and in supporting effective adaptation to teleconsultation.

**Conclusions:**

This pilot project represents a significant step forward in improving care for CHSLD residents in Quebec. Teleconsultation not only makes it possible to overcome recruitment challenges and ensure the continuous presence of nurses during night shifts but also optimizes professional practices while ensuring the safety and quality of care provided to residents.

## Introduction

### The Health Care Situation for Older Adults in Quebec

The health care landscape in Quebec, particularly for older adults, presents numerous challenges and opportunities for improvement. Health and social services professionals work in an environment that is both demanding and constantly evolving. Thanks to scientific progress and medical advances, Western populations, including those in Quebec, are enjoying increased life expectancy. According to the latest demographic data, Quebec is facing a significant aging of its population, and approximately 25% will be considered older within the next decade [1]. This demographic shift creates specific health care needs, particularly in settings, such as residential and long-term care centers (Centres d’hébergement et de soins de longue durée; CHSLDs), where vulnerable populations often require extensive care and support. However, increased longevity often comes with multiple comorbidities and chronic conditions that necessitate holistic care approaches addressing physical, psychological, social, and functional needs [2].

For the past several years, the Quebec government has implemented programs enabling older adults to remain at home for as long as possible [3]. Maintaining older adults’ autonomy in their own living environment reduces pressure on health care facilities while providing personalized, high-quality care [4]. However, older adults living in CHSLDs often experience chronic illnesses that lead to significant disabilities and severe functional limitations [5]. It is estimated that 3% to 4% of Quebecers aged ≥65 years reside in CHSLD [6]. The main reasons why older adults move to long-term care facilities include neurocognitive disorders, chronic pathologies, severe degenerative diseases, and mental health problems [6,7]. These particularly susceptible residents require rigorous attention from multidisciplinary care teams.

### The Role of the Nurse and Nursing Assistant in CHSLDs

Nurses play a central and essential role in Quebec’s CHSLDs [8]. To become a nurse, candidates must complete college- or university-level education and successfully pass a professional examination regulated by the Order of Quebec Nurses (Ordre des infirmières et infirmiers du Québec; OIIQ). Nurses ensure a continuous presence for residents, assess their physical and mental condition, develop therapeutic nursing plans (TNPs) to coordinate interventions, and prescribe treatments with the approval of their professional regulatory body, under defined conditions. The OIIQ stresses the importance of nurses’ contribution to CHSLDs, across all shifts, given the residents’ fragility and the growing complexity of clinical situations [9].

In addition to nurses, nursing assistants also play a fundamental role in CHSLDs. They complete a 2-year vocational training program and must pass a professional examination regulated by the Order of Quebec Nursing Assistants (Ordre des infirmières et infirmiers auxiliaires du Québec [OIIAQ]). Working closely with the nurses, they take part in the entire care process within their professional scope of practice [10]. Their interventions are based on verbal or written medical orders, the TNP, organizational protocols, and the rules governing care. The renewed position of the OIIAQ highlights the nursing assistants’ skills and the significance of their contribution to the quality of care and services provided in CHSLDs.

In Quebec, CHSLDs are integrated within regional health and social service structures. The management of these facilities is overseen by local administrators and supported by a range of clinical managers. Multidisciplinary teams work in CHSLDs, but the residents’ primary needs are largely met by professional nursing teams and personal support workers.

A key aspect of nursing roles in CHSLD is the dynamic collaboration between the nurses and the nursing assistants. The work environment in CHSLDs often features a strict hierarchy, where decisions are shared using a top-down approach [11]. This hierarchical structure is reflected in the collaboration between the nurse and the nursing assistant, which is generally characterized by a supervisory relationship [12]. This type of hierarchy can generate tensions, undermining collaboration [13] and professional identity [14]. Because of their heavy workloads, nurses and nursing assistants rarely have time to exchange ideas, collaborate, and organize the division of tasks [11]. Nevertheless, when they are able to work collaboratively, the partnership between the nurse and the nursing assistant in CHSLDs is particularly beneficial [15,16]. Such collaboration fosters better professional communication [17] and high-quality care for residents [18].

### Workforce Challenges in CHSLDs and Implications for Resident Safety

In Québec, the shortage of nursing staff is particularly acute in rural areas, where attracting and retaining personnel is a persistent challenge. Long-term care is often perceived as an unattractive and difficult sector [7]. Consequently, CHSLDs have experienced staff shortages for several years, a situation that was exacerbated further by the COVID-19 pandemic. To address this, various initiatives have aimed to improve the perception of working in CHSLDs [6]. In the meantime, administrative solutions, such as employing independent workers, overtime, or on-call telephone services, have been used to meet staffing needs. Despite the financial incentives for nurses to take night shifts, this work schedule remains unattractive due to its impact on social [19] and personal health [20]. This is particularly problematic in rural areas, where the availability of nursing expertise is even more limited during night shifts.

The literature indicates that a considerable number of adverse events occur during the night in CHSLDs [21]. Without an on-site nurse, staff are forced to transfer residents to hospital emergency rooms to obtain the care they need [22]. Yet, if there are not any unexpected incidents, the skills of night nurses are often underused. They end up performing support tasks that could be carried out by other professionals, such as nursing assistants or orderlies [23]. Although it is frequently considered as a way of overcoming the challenges of recruiting care staff, replacing a nurse with a nursing assistant in an older adult’s residence amounts to a halfway solution [17]. Researchers agree on the relevance of reviewing the management of time dedicated to care [24], adopting mechanisms to optimize professional expertise [6], and improving care by exploring innovative approaches that are tailored to the CHSLD environment [11].

### The Potential of Teleconsultation in CHSLDs

Faced with challenges that potentially compromise the quality and safety of resident care in CHSLDs, the Quebec government introduced a teleconsultation pilot project for small, rural CHSLDs. This initiative, the first of its kind in the province, aimed to provide web-based access to nurses’ expertise during night shifts when nursing assistants were present at residents’ bedside.

Inspired by on-call telephone service practices, this initiative enabled nursing assistants to consult a nurse by regular phone calls when a resident experienced a health issue. The teleconsultation pilot project formalized improved nursing practice by integrating organizational and technological innovations.

The deployment of the pilot project was carried out in accordance with strict criteria established by Quebec’s Ministère de la Santé et des Services sociaux (MSSS). The project sought to enhance the quality of care provided to residents while ensuring that nursing expertise was readily accessible, even in the most challenging circumstances. Teleconsultation is based on a new organization of care, requiring that institutional documents governing the practices of nurses and nursing assistants be updated, while respecting their separate scopes of practice. The addition of technological tools enables the nursing assistant, who is on-site at the CHSLD, to benefit from the expertise of a remote nurse in real time when a resident’s health situation requires it. To develop this practice effectively, the innovations must work together to meet the needs of all stakeholders [25]. The teleconsultation project uses the Microsoft Teams platform, with nursing assistants using tablets and nurses using laptops, making real-time communication possible.

Although telehealth practices are already well established in long-term care facilities internationally [26], they are yet to be adopted in Quebec CHSLDs. The literature shows that teleconsultation improves access to health care and services when they are needed while optimizing coverage by health care professionals beyond business hours [27-29]. In addition, residents benefit from quick and easy access to an in-depth assessment by a competent health care professional [30].

One of the critical reasons for focusing on small CHSLDs is the chronic shortage of health care staff, particularly during night shifts when nursing expertise is limited. By leveraging teleconsultation and IT tools, this pilot project helps address these workforce issues, allowing small CHSLDs to provide timely and appropriate care even with reduced on-site staff. Teleconsultation offers a solution to help mitigate the ongoing challenges related to recruitment and retention, particularly in rural settings where staffing is a persistent issue. The integration of teleconsultation enhances health care accessibility and helps ensure continuity of care for residents, thus reducing the burden on overworked personnel.

Successful implementation of teleconsultation services relies on close collaboration between the players involved, healthy relationships [31], and intuitive IT systems that support access to all data relevant to the remote care management of residents [32,33]. Thus, it is important that we study the rollout of the nursing teleconsultation pilot project in small CHSLDs and its impact on the professional practice of nurses and nursing assistants.

### Objective

The aim of this study is to explore nurses’ and nursing assistants’ experience of the integration of teleconsultation during night shifts in rural Quebec CHSLDs with ≤50 beds.

## Methods

### Study Design and Setting

The 6-month pilot project was rolled out in 3 rural CHSLDs located in 2 administrative regions of Quebec. The regions were selected by the MSSS for the alignment with the project’s outlined criteria, which include facilities situated in semiremote and remote areas, those already experiencing nursing shortages during the night shift, and those reporting issues and risks related to the shortages. In addition, at least 30% of all CHSLD facilities in the territory have a capacity of ≤50 beds. The rollout was conducted sequentially from July 2022 to March 2023 at different sites. During the project, participants documented 19 clinical situations using teleconsultation and 14 clinical situations without teleconsultation. According to the organization’s previous year’s statistics regarding on-call telephone services, the number of clinical situations remains similar year over year. This attests to the stability of the environment, providing an appropriate context to study the implementation of teleconsultation. Given the innovative nature of the pilot project, an exploratory qualitative study was conducted to understand the impact of implementing teleconsultation in nighttime nursing care.

### Data Collection

In total, 2 interview guides were designed, tested, and validated by the research team. The first guide, comprising 12 open-ended questions, aimed to identify the barriers and facilitators to the implementation of teleconsultation. This guide provided a better understanding of the context and experiences surrounding the pilot project’s deployment. The study was guided by key factors influencing the implementation of health innovations, as outlined in the framework proposed by Chaudoir et al [34], along with the Consolidated Framework for Implementation Research [35]. The second guide, including 12 open-ended questions, focused on assessing the impact of newly introduced workflows on nurses and nursing assistants in the context of teleconsultation. This guide was based on a comprehensive literature review associated with nursing roles [36], the use of nursing resources [37], and the scope of professional practice [38]. According to the findings, 3 distinct levels of nursing practice were identified—clinical, administrative, and organizational—allowing a thorough assessment of the contributions made by both nurses and nursing assistants. By using both interview guides, the research provides a more holistic and nuanced understanding of the phenomenon under study. The interview guides are included in Multimedia Appendix 1.

In addition, a web-based sociodemographic data collection form, created on the Google Forms platform, was also shared with and completed by participants, enabling the collection of information about their current employment, work experience, and academic background. Participants were contacted by email, which included the research consent form, interview guides, and the project poster. The consent form explained the context, project objective, procedure and duration, anticipated benefits, and anonymity and confidentiality.

### Participants

Participant recruitment was done using nonprobability sampling [39], through which participants were identified by pilot project managers. To be eligible, participants had to be nurses or nursing assistants, have a formal employment relationship with the organization, and have used teleconsultation during the 6 months that it was deployed in the CHSLDs. A total of 8 nurses and 7 nursing assistants were contacted by the research team between March and July 2023. In total, 62% (5/8) nurses and 57% (4/7) nursing assistants consented to participate. The remaining individuals did not respond to the email invitation.

Pursuant to an agreement with the Direction nationale des soins et services en soins infirmiers of the MSSS, the semistructured interviews were conducted during the participants’ working hours or, if this was not possible, participants were compensated for the time devoted to the interview according to the conditions of their work agreement. Regarding this directive, only 22% (2/9) of the participants conducted the interviews during off-hours.

A total of 2 semistructured interviews, each with a duration of 60 minutes, were conducted in French. Of the 9 participants, 3 (33%) were interviewed via videoconference (Zoom; Zoom Video Communications, Inc), and 6 (67%) were interviewed by phone, according to each participant’s preference. The main researcher (VN) conducted the interviews associated with the first guide, drawing on her previous experience in this specific field. In contrast, the coresearcher (MCL) conducted the interviews associated with the second guide due to her nursing qualifications and knowledge of the profession. All interviews were audio recorded with participants’ permission, and then transcribed in compliance with ethical and confidentiality standards. The deidentified recordings were transcribed verbatim by a third-party transcription service bound by a confidentiality agreement. Participants were given the option to review the transcripts of each interview, but none of the participants chose to receive the transcripts.

No additional recruitment process was necessary, as information redundancy indicated data saturation [40]. Each participant completed 2 semistructured interviews, resulting in a total of 18 interviews conducted with 5 nurses and 4 nursing assistants between March and July 2023. The participants had no personal or professional relationship with any members of the research team.

### Data Analysis

Interview data were processed using NVivo software (version 14; Lumivero). Each interview was transcribed verbatim immediately after it had been conducted and analyzed by the research team. Validation and reflexivity steps were integrated throughout the analysis process to ensure compliance and authenticity of the approach.

Before beginning the analysis, we planned to structure the data using a 3-level framework (clinical, administrative, and organizational), as it aligned with our understanding of nursing and nursing assistant practices. This framework was supported by relevant literature [36-38]. The clinical level relates to the care provided directly to residents, the administrative level concerns the organization of interventions, and the organizational level focuses on innovative initiatives.

Initially, the research team familiarized themselves with the data by reading and rereading the transcripts to gain an in-depth understanding of the content. This immersive experience confirmed the suitability of the 3 levels of the framework and revealed interactions between the levels, such as how the organizational level influences the administrative level, which in turn impacts the clinical level.

Following familiarization, the team engaged in a systematic coding process, identifying key themes and categories that emerged from the data. The coded data were then regrouped into categories that reflected the interactions among the 3 levels of practice. In addition, participants’ lived experiences and perspectives regarding the deployment of the pilot project were categorized as either hindering or facilitating factors. The directed approach to qualitative data analysis, as used here, aligns with previous research aimed at better understanding nursing practice and its impact [41].

Workflows, inspired by lean methodology, played a central role in understanding the practices of nurses and nursing assistants. These workflows ensured the smooth flow of operations to improve performance and create value [42]. The clinical approach to nursing encompasses tasks such as data collection, analysis, planning, intervention, and outcome assessment related to the care and services provided directly to residents. The administrative and organizational levels of nursing practice have an overarching influence on these workflows, serving as the foundation for clinical activities.

The integration of teleconsultation impacted nurses’ and nursing assistants’ practices in various ways. The research team categorized key findings at each level of practice based on their impact on workflows. Enhanced practices added value to workflows by improving the quality of interventions, while unchanged practices demonstrated an inherent stability in CHSLDs despite the integration of this new technology.

To finalize the analysis process, data organization and interpretation allowed the research team to identify 3 major underlying themes: leadership, collaboration, and impact on residents.

### Ethical Considerations

Ethics approval was obtained from the research ethics committee of the Outaouais Integrated Health and Social Services Centre before the beginning of the study (reference number 2022-353_195), in Quebec, Canada. All participants gave their consent electronically before beginning the survey. Participation was anonymous and voluntary. The privacy rights of the study participants were observed. The study participants did not receive monetary compensation. The study’s findings will be disseminated through presentations at conferences and publications in peer-reviewed journals using anonymized data. Findings will also be shared through presentations to various MSSS stakeholders and the nursing community.

## Results

### Characteristics of Participants

The sociodemographic data collection form provided a brief portrait of the study participants. All 9 participating interviewees completed the form. In total, 5 (56%) of the participants were nurses and 4 (44%) were nursing assistants. Of these participants, 7 (78%) worked full time, while 2 (22%) worked part time. In terms of educational background, 3 (33%) participants had a vocational diploma, 2 (22%) had a college diploma, and 4 (44%) had a university degree. It is important to note that, in Quebec, it is possible to become a member of the OIIQ and enter the nursing profession with a college or university degree.

The participants’ experience of working in CHSLDs varied: 33% (3/9) of the participants had been working there for <5 years, 33% (3/9) had between 5 and 10 years of experience, 22% (2/9) had between 11 and 20 years of experience, and 11% (1/9) participant had >21 years of experience. According to the literature, a nurse with ≥5 years of experience in a specific field can be considered an expert [43].

The integration of teleconsultation into their professional practice led to a variety of responses and experiences for participants. Their diverse reactions highlighted the benefits and challenges associated with implementing new technology in long-term care. The subsequent sections present nurses’ and nursing assistants’ perceptions of the impact of teleconsultation on the 3 levels of their practice examining areas where it created unchanged practices, demonstrating stability, and where it introduced enhanced practices, enabling the illustration of optimized workflows when using teleconsultation.

### Unchanged Practices

Unchanged practices reflect an inherent stability within CHSLDs, even with the integration of teleconsultation. This stability indicates that certain fundamental aspects of care delivery and professional interactions have remained consistent, despite the introduction of this new technology.

#### Organizational Level

Participants were asked to share their views on their level of involvement in planning the pilot project. Most (8/9, 89%) of the participants agreed that they had been presented with well-defined, predeveloped content at the teleconsultation information meetings. This approach came as no surprise to participants, who said they were satisfied with this aspect:

Everything was already in place. Basically, they worked together, the manager, the project manager, and our administrative officer. When we first heard about the project, everything was already in motion, it was already set up. The binders were already made. The step-by-step instructions were already inside. All our protocols too. I’d say we didn’t really participate. We just kind of stepped into the project, then set it in motion, that’s all. We weren’t consulted beforehand.

Participant 6

However, the nurses and nursing assistants did say that they wished they had been involved in the initial reflection and decision-making on the pilot project:

Well, I could have shared my opinion, and some ideas too.

Participant 3

I’d say that everything was really well structured and complete. That’s why I didn’t have anything to add. But of course, if they had asked my opinion, I would have wanted to participate, of course, to share my point of view.

Participant 6

In this regard, participants shared some of the issues associated with the planning. They expressed dissatisfaction with the time frames determined by the managers:

We had time, but it kind of dragged on at some point. You know, you tell yourself: Well, are we going to do it or not? When are we starting? We’ve all been trained. It’s been three months, but nothing is happening yet. That’s why, you know, I thought it took a long time to get started.

Participant 2

It was pretty drastic. It was like “OK, you’re getting teleconsultation training...” Uh, what’s that? We were kind of thrown into it, and we were a bit taken aback.

Participant 7

Finally, participants wished they had had ongoing support throughout the pilot project, not just at the start:

The project was launched and we just went on from there. Then, there was no real support from the manager.

Participant 6

Participants highlighted a relevant point related to professional maturity, experience, and ease. Having significant work experience contributes to the enrichment of professional practice every day, not just within the context of the pilot project. Professional maturity, in the nursing field, refers to the ability to apply clinical judgment with confidence, adapt to complex situations, demonstrate autonomy in decision-making, and help improve the interventions carried out with residents:

Maturity, yes, that...well, there’s training and all that, but there’s not just that, there’s life experience. If I’d been a new nurse, I’d have been very uncomfortable. After all, I have over twenty years’ service.

Participant 1

Prior to the teleconsultation program, even a few years ago, nursing assistants were already covering the nighttime shifts. Then, at some point, they abolished that, and decided that they needed a nurse on duty 24-7. So, you know, the rest of us were already used to it, because I’ve always worked nights, so I was used to the process. So for me, it wasn’t anything new.

Participant 4

#### Administrative Level

Participants discussed the various aspects of working collaboratively, including documentation in the resident’s medical record. The experiences suggest that the rules of documentation were not implemented in a standardized way. Some participants documented too much, others just enough, while some opted to enter information later. However, according to 1 participant, this phenomenon had already been observed before the implementation of teleconsultation:

In the morning, the nursing assistant would tell me: “I gave her a Tylenol, she had pain in such and such a place. It was already in the TNP, so I didn’t need to consult on this.” Indeed, it was fine, but I would, let’s say, write my note, then enter the nighttime call for such-and-such a case, confirm with the TNP, and everything, so it was fine.

Participant 1

There’s a lack of information being shared, but that, as I say, is not just because of teleconsultation, not at all. It’s a generalized issue.

Participant 8

Another unchanged element, at the administrative level, is the importance afforded to residents’ families and loved ones. The nurses and nursing assistants maintained a special focus on this relationship, upholding the same values and principles:

It hasn’t changed the practice. That’s why this aspect hasn’t been integrated as such. We’ll talk about it with the family, but really, you know, the “We’re now using video teleconsultation” aspect, well, there were people who weren’t even aware of the fact that there wasn’t a nurse on site during the night.

Participant 6

Well, Mom fell last night. I was the one on duty, and then I took care of her. They won’t ask me if I used teleconsultation or not. They just need to ask me if there are any after-effects, if there’s anything wrong.

Participant 2

#### Clinical Level

Participants highlighted a number of limitations associated with a virtual assessment of residents’ physical and mental condition. As with on-call telephone services, certain practices, such as palpating residents and managing certain clinical situations, are incompatible with teleconsultation:

I’m actually someone who likes to move around, who likes to see things in real life to validate certain information, to palpate, you know, to conduct a fuller assessment.

Participant 6

Many, many of our residents suffer from cognitive decline and unfortunately they don’t understand. They didn’t understand the instructions; they don’t understand when we speak to them. They can’t see us in the camera. You know, because it’s nighttime, they’re often sleepy as well. It’s not always easy.

Participant 1

Despite the integration of teleconsultation, the sense of responsibility, clinical judgment, and desire to provide safe, high-quality care and services to residents remains unshaken. Teleconsultation has not changed the values and principles of nursing assistants:

My decision-making would probably have been the same, with or without teleconsultation, once I came here. It didn’t change my decision-making.

Participant 2

I’m still part of the same order, I still have the same scope of practice to uphold. So, from my standpoint, my responsibilities remain the same. Just because you have a tablet doesn’t mean you’re protected. No, it gives us an additional tool, but we still have to use our judgment.

Participant 8

### Enhanced Practices

Enhanced practices refer to the added value brought to workflows by improving the quality of interventions. These advancements not only streamline processes but also elevate the standard of care provided, resulting in more effective and responsive support for residents.

#### Organizational Level

Participants praised the rigorous planning of the pilot project and the quality of training. Presentations were clear and the content was well documented:

I think the training was well done; we were well trained.

Participant 2

In addition, the sessions dealt with updating and creating clinical tools tailored to teleconsultation:

For example, we didn’t used to have algorithms. That was something new with teleconsultation.

Participant 4

We updated all our protocols, you know, to raise awareness. We updated “Are the collective prescriptions correct?” So, you know, we did a good job of updating, which should be ongoing, but which, well, isn’t necessarily done as part of day-to-day activities.

Participant 5

In addition, the training sessions prompted group discussions on professional roles, professional order guidelines, understanding the scope of practice, and recognition of the expertise of other disciplines:

It’s when we did the training with the project officer. Well, with the eligibility criteria, you know, exclusions and so on. We realized that, oops, sometimes our nursing assistants were being very autonomous. We’ve now rectified that. I think we adjusted well after that.

Participant 6

Inevitably, with everything being more up to date like this, I don’t need to communicate with my nurse as much. I’m more independent.

Participant 7

In addition, participants appreciated the attention paid to their concerns and the opportunity to provide immediate feedback during the training sessions:

We were asked if there were things we thought should be added, things that were missing.

Participant 9

But more so in connection to, say, the material we put in the case, the forms, the step-by-step instructions. Yes, we were consulted. We were asked if there were things they thought we should add, things that were missing. They were also available if we wanted to add anything along the way.

Participant 8

#### Administrative Level

Teleconsultation enabled the remote nurse to contribute to the coordination of interventions in real time and adjust the resident’s medical record. For example, the remote assessment of a resident’s physical and mental condition was immediately documented in the TNP, enabling the nursing assistant to intervene according to her scope of practice when the health situation changed unexpectedly:

Well, with teleconsultation, I changed an TNP remotely...with this tool, we’re able to directly change things remotely.

Participant 5

The fact of sending prescriptions, the fact of sending notes, of sending everything directly to the home, I find that it...How can I say this? It decreases the risk of errors in the end.

Participant 8

Another element mentioned by participants was predictability. Because the pilot project was formalized, supported, and recognized by the organization, nurses and nursing assistants knew in advance when teleconsultation was taking place and who would be involved. This enabled better care planning, made it easier to anticipate potential needs, and gave nurses and nursing assistants a greater sense of security:

If I see that the patient...I know that in two hours his third dose will be due, then I’m going to get organized.

Participant 1

Some participants noted a marked improvement in the design and writing of the TNP, believed to be attributable to a growing awareness of the need to optimize the nursing assistants’ scope of practice. In the context of nursing teleconsultation, the TNP is written jointly by the nurse and the nursing assistant to target residents’ health problems efficiently. The nursing assistant exercises full autonomy when updating the TNP, adapting it to the resident’s health-related condition and needs, and proposing relevant interventions:

I think this process, with the night nurses, forces us to have nice TNPs. So, you know, it may have been...That was during our first calls. But shortly after, we got back on track.

Participant 5

We also established plans, well, our TNPs. We really updated them to enable [nursing assistants] to be autonomous.

Participant 6

#### Clinical Level

Participants noticed that the pilot project helped improve the professional bond between the nurse and the nursing assistant. During the calls, a knowledge-sharing and coaching process was established between them. The nurse ensured a real-time virtual presence for the nursing assistant, guiding them, suggesting interventions, and supporting their practice:

You know, we were able to work a bit as a team there. In the sense that, you know, she could suggest things that I could pay more attention to.

Participant 3

It means I can talk to my nursing assistant. Then, it’s like we’re really comfortable; it’s like we’re together. I think that makes it easier to conduct the interventions.

Participant 8

The rollout and updating of the pilot project–related clinical tools clarified professional roles and fostered the achievement of full autonomy with confidence. For example, the addition of decision-making algorithms, improved TNP writing, and adjustments to nursing protocols built nursing assistants’ confidence and contributed to their empowerment. This process facilitates decision-making, promotes the development of clinical judgment, and improves professional ease:

For example, we didn’t used to have algorithms. That was something new with teleconsultation. Yes. When there’s a situation that’s a little ambiguous, you feel like you’re sitting on the fence. Then you’re not sure which side to choose. Well, you follow your algorithm and it leads you straight to the correct way of doing things.

Participant 4

I’d say it’s generally going well. I haven’t experienced any ambiguous situations. The thing is that teleconsultation can only make it even better. I think the fact of talking to and also seeing your nursing assistant...I think it can only bring positive elements in that regard...being able to exchange with them as such.

Participant 6

Finally, participants stated that teleconsultation enhances remote nursing by enabling visual inspection. Assessing the physical and mental condition of a symptomatic resident is the responsibility of the nurse, requiring professionalism, diligence, and conscientiousness. Teleconsultation provides valuable visual support to nurses, enabling them to observe clinical situations directly, make more accurate assessments, and monitor the evolution of the resident’s health condition. Furthermore, the addition of this tool also benefits the nursing assistant, fostering more enriching discussions with the nurse and enabling a better understanding of the situation, thus refining the chosen intervention:

For me, teleconsultation using a tablet was a practical way of getting a visual. It helps justify a problem. Then, afterwards, once you have visualized the problem, you can find more solutions to solve it. So I found that it was like an additional tool to support and reinforce my role and responsibilities.

Participant 4

We have a lot of patients who are agitated and aggressive. This video aspect is good, because I can see in real time what the patient is doing, without having to go there myself. It’s a situation. Otherwise, in the case of a wound, you know. You can see it.

Participant 6

Table 1 summarizes the unchanged and enhanced practices for each level of practice.

**Table 1:** Enhanced and unchanged practices by level.

Level of practice	Enhanced practices	Unchanged practices
Clinical level	Collaborative team dynamics during teleconsultation, where knowledge is shared between remote nurses and nursing assistants, reinforcing their respective professional roles Remote nurses’ clinical decisions are reflected in the TNP^a^ in real time Addition of visual support to improve data collection, situation analysis, and intervention implementation	Palpation remains impossible when using teleconsultation Certain clinical situations make teleconsultation-based assessment difficult Professionalism is ensured in the interest of providing exemplary care
Administrative level	Improved clinical communication enabling the remote nurse to make additions to the medical record Proactive drafting of the TNP to reinforce collaboration between remote nurses and nursing assistants for optimal professional practice	Resident medical record documentation rules are not implemented in a standardized way Communication is maintained between nursing professionals and residents’ families and loved ones
Organizational level	Openness and availability of project managers to provide information at the start of the project Complete and tailored training program Updated protocols, rules governing care, collective prescriptions, and additional clinical tools Enhanced scope of practice-related knowledge	Professional nursing team members not solicited enough during the planning phase of the pilot project—change management Professional experience and maturity are essential to rolling out teleconsultation

^a^TNP: therapeutic nursing plan.

## Discussion

### Principal Findings

#### Overview

**Figure 1:**
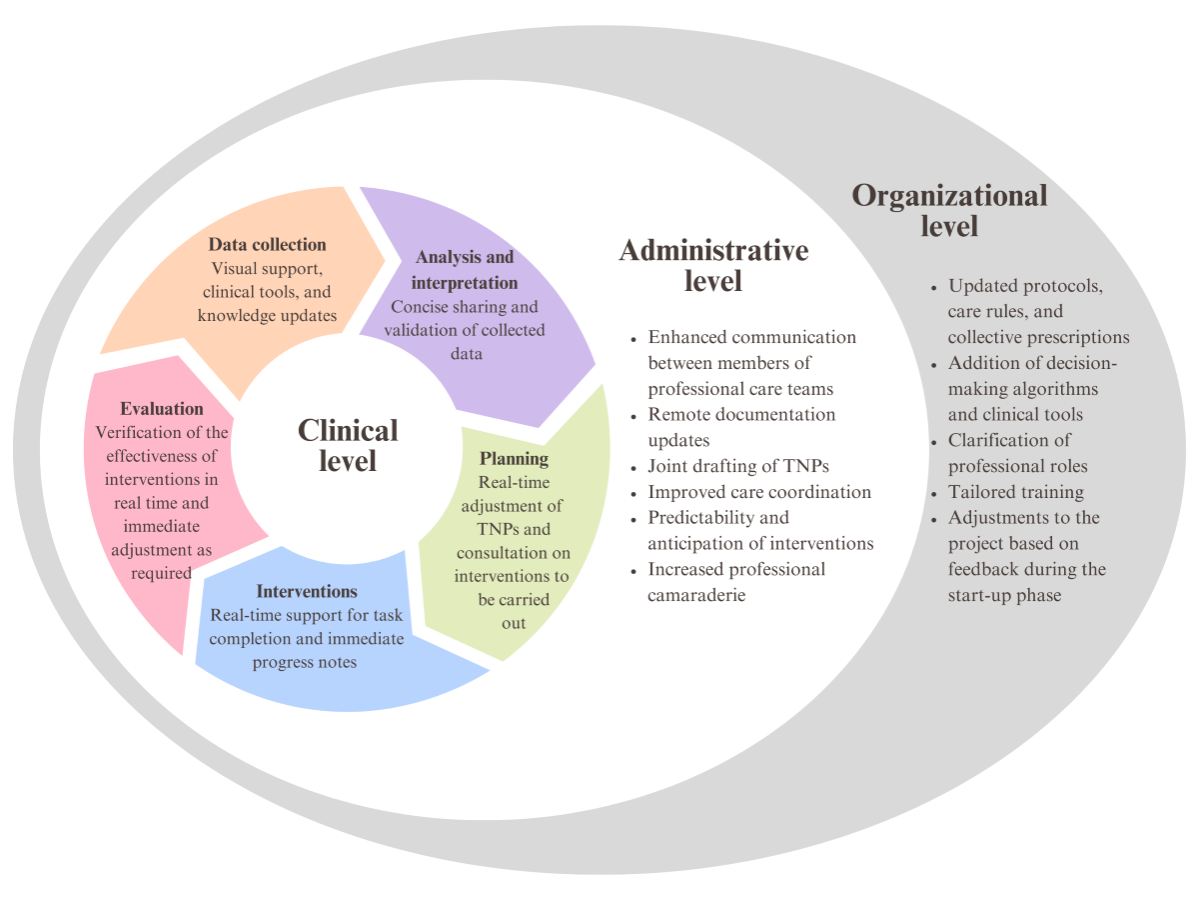
Workflows optimized by teleconsultation in nursing. TNP: therapeutic nursing plan.

The implementation of teleconsultation elicited a diverse range of responses from participants, revealing both unchanged and enhanced experiences. These findings underline the benefits of adopting new health care technologies. The nurses’ and nursing assistants’ perceptions highlighted several positive aspects where teleconsultation introduced beneficial changes. Figure 1 illustrates the workflows optimized by teleconsultation in nursing and shows the interactions between different levels of practice and their associated benefits. The organizational and administrative levels supported clinical practice, which is represented by the clinical approach in nursing. From planning to assessment, each step benefited from the integration of technology and increased collaboration within the care team. Real-time documentation, visual support, role clarity, and proactive communication ensured seamless continuity of care, with an emphasis on efficient and safe interventions.

However, the experience also raised concerns about administrative management, change management, and compliance with documentation rules. Nevertheless, most (8/9, 89%) participants enjoyed the structure and quality of the training courses as well as the clarification of professional roles and the opportunity to update and implement clinical tools tailored to teleconsultation. Participants did not report any scope creep during the pilot project.

Although teleconsultation enhanced certain aspects of nursing practice, namely by facilitating care coordination and boosting professional confidence, it also required adjustments to optimize integration. These findings underline the importance of appropriate support and careful planning to maximize the benefits of such technological innovations in long-term care settings.

The qualitative findings will be discussed around 3 cross-cutting topics. It is important to note that they emerged from the data analyses: leadership, collaboration, and impact on residents.

#### Leadership

##### Overview

Nurses’ and nursing assistants’ workflows were improved through an extended scope of practice, which can be attributed to the leadership exercised during the teleconsultation project. Interventions and discussions initiated by project managers clarified scopes of practice, eliminating ambiguities, and building the confidence of nurses and nursing assistants [44]. This led to an increased commitment from participants and extended the scope of practice. The literature has highlighted that clinical leadership can be exercised in a caring way to improve the quality of interventions with residents [45]. The OIIAQ’s recent work to strengthen the role of nursing assistants [46] is embodied by this teleconsultation pilot project, demonstrating how a supportive environment can promote optimal practice [47].

Nurses’ leadership is also linked to their experience and professional maturity. The leadership exercised by an expert nurse leads to positive results for residents and care teams alike [45,48]. Indeed, most (8/9, 89%) interviewees had >5 years of experience working with older adults, and the literature supports the view that expertise contributes to the success of teleconsultation projects [49,50].

##### Shared Governance

Shared governance is an important concept for the development of high-level leadership among nurses and nursing assistants. Researchers have pointed to a gap between managers’ and nurses’ vision, sometimes making it difficult for these 2 groups to communicate [51]. During the pilot project, participants were not always asked about their perspectives, leading to dissatisfaction with the planning and the intensity of follow-up.

Shared governance, involving nurses and nursing assistants from the outset of the pilot project, could have improved the planning of health care projects, refined the prioritization of initiatives, and promoted ongoing improvement [52]. Moreover, it would enable project managers to better meet the technology-related needs and expectations of end users: the nursing staff [53].

#### Collaboration

##### Overview

Nurses’ and nursing assistants’ workflows were improved by strengthening their collaborative relationships. Teleconsultation consolidated these relationships, leading to optimized care planning, intervention coordination, and access to clinical documentation.

First, teleconsultation enabled nurses to share information with the nursing assistant and complete clinical documentation in real time, thereby reducing the number of interpretation errors. This synchronous and asynchronous communication ensured better continuity and coordination of care [54]. The care team was able to access progress notes, prescriptions, and the TNP as required [55,56]. However, despite these benefits, there were issues related to the implementation of documentation. Although documenting interventions is a professional obligation [57], studies have shown that nursing notes are sometimes superficial, affecting the quality and continuity of care [58]. This highlights the importance of integrating users, processes, and technology [59,60].

Second, the collaborative dynamic between nurses and nursing assistants was strengthened by a clear definition of their roles. Project managers played a decisive role in optimizing the use of people’s skills [44]. However, professional development remains a contextual phenomenon, influenced by local and organizational factors [61,62]. By harmonizing nurses’ and nursing assistants’ contributions, they fostered collaborative practice and improved communication within teams [63].

Finally, team stability is crucial to strengthening collaborative relationships. It helps establish a climate of trust between colleagues, which is essential to ensure optimal professional practice [64]. The pilot project demonstrated that collaboration, underpinned by effective consultation, enables the achievement of targeted objectives and the provision of care tailored to residents’ needs [65].

##### Predictability

Teleconsultation improves schedule predictability, an important consideration for nursing staff [66]. Clearly identifying the schedule or timetable of night shifts, during which telecommunication would be used, facilitated collaboration between nurses and nursing assistants, enabling them to anticipate and respond proactively to residents’ needs. For example, a jointly drafted TNP improves care management for residents with recurring health problems [57]. The nursing assistant can then rapidly carry out the necessary interventions safely and with confidence.

In addition, teleconsultation reduces the need for mandatory overtime, preserving nurses’ physical and psychological health [67,68]. Teleconsultation makes it possible to organize required interventions in advance to ensure the continuity and safety of care [69].

#### Impact on Residents and Their Loved Ones

##### Overview

Interviews with nurses and nursing assistants revealed that the well-being of residents, as well as that of their families and loved ones is a constant and central concern. Every intervention, whether or not it includes the use of teleconsultation, must be of impeccable quality, ensuring the well-being of residents at all times. Therefore, it is obvious that when using teleconsultation, nurses’ and nursing assistants’ workflows must establish the resident as the top priority.

##### Clinical Aspect of Practice

Participants demonstrated unwavering professionalism and respect for professional values when using teleconsultation. They recognized both the benefits and limitations of the technology. During complex clinical situations, they intervened with greater confidence and ease. Because it involves the use of a camera, teleconsultation facilitates the physical assessment of a resident’s condition. However, participants were also aware of situations where teleconsultation was less suitable and recognized the imperative need to comply with the professional and ethical standards in remote practice.

The amount of literature on integrating technology into health care is growing rapidly [70]. Recent initiatives on remote clinical monitoring [71] have led to the publication of nursing practice standards that also consider technological practices [50]. Participants agreed that teleconsultation is a useful additional tool but that it can never replace a full physical examination or direct interaction with the resident. A similar case study on teleconsultation also concluded that this modality of care does not replace the presence of a nurse with the resident but can effectively support clinical practice when the situation allows [72]. In short, pilot project participants demonstrated a strong commitment to and rigorous professional integrity in providing safe, high-quality care to residents.

##### Involvement of Family and Loved Ones

The active presence of residents’ families and loved ones in the organization of care and services in CHSLDs is not only desirable but also essential. The teams who took part in the pilot project set up a communication plan to inform residents’ families and loved ones of this initiative, encouraging them to ask questions as needed. Nurses and nursing assistants then discussed the specifics of the pilot project when obtaining consent for the residents to participate, providing an opportunity to clarify certain aspects, to chat with their families and loved ones, and to reinforce existing bonds of trust.

The literature on this subject supports the participants’ observations. Introducing the technology-based project to residents’ families and loved ones and obtaining their formal consent is essential [71]. Strengthening the relationships between care staff and the residents’ families and loved ones is highly beneficial. The integration of technology in long-term care facilities is now widely accepted [73] and promotes the involvement of residents’ loved ones in clinical decisions [74].

Finally, this integrated approach to leadership, collaboration, and resident-centered health care in a teleconsultation context illustrates how these elements depend on each other to optimize practices and improve clinical and organizational outcomes.

### Limitations and Future Research

Despite the rigorous methods used as part of the study, certain limitations must be considered to improve the interpretation of the results and more accurately target the prospects for teleconsultation in long-term nursing care during night shifts. First, the pilot project was conducted in 2 Quebec regions, it involved 3 rural CHSLDs, and the sample size was limited to 9 participants. However, it is important to note that these participants used teleconsultation for 6 months, logging 19 clinical situations, a number similar to data collected by the CHSLDs over the same period in the previous year. This exploratory study was conducted in small care settings, where care teams are limited by the number of residents, justifying the scope of the study. Nevertheless, caution is paramount when generalizing the results to other regions or similar contexts.

To broaden our understanding and increase the effectiveness of teleconsultation in long-term nursing care during night shifts in small, rural CHSLDs, several avenues of research may be considered. Expanding this research to a larger number of small CHSLDs in different regions would improve the representativeness and generalizability of the results. A larger sample would more accurately capture the context-specific and organizational variability.

In addition, a comparative analysis of different geographical and organizational settings would be relevant to identify the factors that influence the success of teleconsultation. This type of analysis would shine a light on best practices and the conditions required for successful implementation.

It would also be worthwhile to assess the long-term impact of teleconsultation on quality of care, resident and caregiver satisfaction, and associated costs. Longitudinal studies could provide valuable data on the benefits and challenges of this approach over an extended period.

Finally, exploring the impact of teleconsultation from the residents’ and families’ point of view would enable us to understand their perceptions, the elements with which they were satisfied, and their concerns. This perspective could provide crucial insights, helping to adjust practices and improve the care experience.

In addition to this study, developing continuing education programs on the use of teleconsultation technology for health care professionals in rural settings could facilitate the adoption of teleconsultation and optimize the use of these tools. Establishing standardized protocols for the integration of teleconsultation into daily care routines could also foster greater efficiency and broader adoption of this technology.

### Conclusions

The implementation of nursing teleconsultation in rural CHSLDs with ≤50 beds has shown promising results in terms of improved workflow, interprofessional collaboration, and the quality of resident care. Nurses’ and nursing assistants’ perceptions revealed tangible benefits such as real-time documentation, increased visual support, and proactive communication. These elements promoted the efficient and safe continuity of care while highlighting the need for adequate support and careful planning to maximize the benefits of this technology.

Their experiences also highlighted challenges such as administrative management, change management, and compliance with documentation rules. Nevertheless, the structured training and clarification of professional roles were widely appreciated, contributing to the successful implementation of teleconsultation without any reported scope creep.

In total, 3 main themes emerged from the data analysis: leadership, collaboration, and impact on residents. Leadership played an important role in clarifying scopes of practice and building nurses’ and nursing assistants’ confidence. Collaboration was enhanced through synchronous and asynchronous communication, enabling better care coordination. Finally, the predictability of schedules and the involvement of residents’ families and loved ones ensured more proactive care management tailored to the residents’ needs.

Although teleconsultation cannot entirely replace the physical presence of nurses, it has been shown to be a valuable tool to support clinical practice and improve the quality of care in CHSLDs. To optimize the integration of teleconsultation, ongoing development of appropriate support and training strategies is essential while also promoting shared governance and the active involvement of residents’ families and loved ones. These measures will help maximize the benefits of this technological innovation and guarantee high-quality resident-centered care.

In short, this study demonstrates that nursing teleconsultation represents a promising technological advance to optimize professional practice and strengthen team collaboration. In light of these findings, it is essential that we carry out in-depth discussions on the future of virtual nursing to better understand and efficiently integrate this practice in long-term care settings.

## Appendix

Multimedia Appendix 1 Interview guides.

## Abbreviations

CHSLD: Centre d’hébergement et de soins de longue durée (French for “residential and long-term care center”)
MSSS: Ministère de la Santé et des Services sociaux
OIIAQ: Ordre des infirmières et infirmiers auxiliaires du Québec
OIIQ: Ordre des infirmières et infirmiers du Québec
TNP: therapeutic nursing plan
