# Supplementary material for: Nurses’ and Nursing Assistants’ Experiences With Teleconsultation in Small Rural Long-Term Care Facilities: Semistructured Interview Pilot Study
Source: JMIR Aging. 2024 Nov 27;7:e65111. doi: 10.2196/65111 (PMC11635311; doi:10.2196/65111)
Supplement: Multimedia Appendix 1 [file aging_v7i1e65111_app1.docx]

**Multimedia Appendix 1 – Interview guides**

**Interview guide session 1**

1. As a participant from CIUSSS MCQ/CISSSCA, what is your role in the deployment of teleconsultation in small residential and long-term care centres (CHSLD)?
2. General question: Since you have been involved in the implementation, how is the deployment of innovations (organizational/technological) in CHSLD going?
3. According to your perception, where do you stand with the implementation of innovations (organizational/technological) in CHSLD?
4. What changes in practice have you observed as part of the deployment in CHSLD?
5. What are your expectations and concerns regarding the implementation of these innovations (organizational/technological)? Now, we will discuss the various favorable elements and obstacles to the implementation of innovations (organizational/technological) in CHSLD.
6. In your opinion, what factors facilitate the implementation of innovations at your site...?
7. In your opinion, what factors hinder the implementation of innovations at your site...?
8. What challenges do you face in your role (nurse or nursing assistant) in the deployment of teleconsultation in CHSLD?
9. Are there any elements that have not been addressed that you would like to discuss?

**Interview guide session 2**

First and foremost, we want to better understand your daily professional activities and the changes brought about by the integration of nursing teleconsultation into your practice.

1. From a clinical perspective, that is to say the interventions deployed with residents, how has nursing teleconsultation modified or not modified your practices?

- Data collection, assessment, analysis and interpretation, implementation of the intervention, evaluation of the intervention
- Preventive practices, reactive practices

1. Regarding the clinical practices mentioned above, can you clarify your perception of your professional responsibilities? Are they the same or different following the implementation of nursing teleconsultation?

- Accountability
- Quality of interventions and actions
- Sense of safety or professional ease

1. Still related to your clinical practices, how has nursing teleconsultation modified or not modified your decision-making process surrounding a clinical situation?

- Assigned role – Role played
- Level of satisfaction

1. In the context of collaborative work among members of the nursing professional teams during a nursing teleconsultation, we would like to hear your thoughts on the complementarity of the expertise associated with professional titles.

- Dependence or interdependence of contributions
- Confusion or ambiguities
- Disciplinary boundaries

1. To conclude this section related to your clinical activities, we would like to know your professional needs surrounding the integration of nursing teleconsultation into your daily practices.

- Training, support, guidance, follow-up, recognition, appreciation

Another aspect that interests us in this study is the various administrative activities that are part of your daily practice. Once again, we would like to know the changes brought about by the integration of nursing teleconsultation.

1. Interprofessional communication as well as coordination of care and services are activities that support clinical activities. What changes have you observed following the implementation of nursing teleconsultation?

- Quality and quantity of information transmitted
- Ease of communication with members of the nursing professional team as well as the interprofessional team
- Interprofessional team meetings
- Ease or difficulty in coordinating interventions

1. We recognize the importance of the contribution of families and loved ones to the various interventions deployed with residents. How has nursing teleconsultation influenced your relationships with the families and loved ones of residents?

- Communication
- Information sharing
- Education

1. The Nursing Therapeutic Plan (PTI) is a tool aimed at improving the coordination of interventions and supporting clinical communication for the benefit of the resident. In your opinion, what changes have occurred regarding the PTI in the context of the deployment of nursing teleconsultation in CHSLD?

- In its completion, its use, its utility

The last aspect that interests us addresses organizational activities. The pilot project for the deployment of nursing teleconsultation in CHSLD mobilized numerous resources and led to the development of the practice of nursing professionals.

1. To this end, what was your involvement in the development of nursing practice in a teleconsultation context?

- Review and modification of care rules, protocols, procedures, forms, etc.

1. The deployment of nursing teleconsultation in CHSLD required training sessions to ensure the implementation of the pilot project. What is your view on the training provided by your organization?

- Involvement and participation in its design/deployment
- Solicitation or not of your expertise
- Opportunity or not to share your knowledge and expertise
- Openness to feedback

1. We would like to know your contribution to the various activities aimed at updating practices as well as developing strategies to improve the quality and safety of residents. In the context of nursing teleconsultation, how do you perceive your input in the previously mentioned activities?

- Level and quality of involvement
- Satisfaction or dissatisfaction regarding involvement (tensions?)

1. Are there any elements that have not been addressed that you would like to discuss?
